# Draft Genome Sequences of Eight *Staphylococcus aureus* Strains Isolated during Foodborne Outbreaks

**DOI:** 10.1128/genomeA.01557-17

**Published:** 2018-02-01

**Authors:** Igor Abaev, Yury Skryabin, Angelina Kislichkina, Alexandr Bogun, Olga Korobova, Ivan Dyatlov

**Affiliations:** a State Research Center for Applied Microbiology and Biotechnology, Obolensk, Russian Federation

## Abstract

We report here the draft genome sequences of eight *Staphylococcus aureus* strains isolated during three large food poisoning outbreaks in the Russian Federation. The strains were collected from clinical specimens and various foodstuff samples.

## GENOME ANNOUNCEMENT

Staphylococcal food poisoning (SFP) is one of the most widespread causes of foodborne intoxication worldwide, and it results from the ingestion of staphylococcal enterotoxins (SEs) that are produced by some *Staphylococcus aureus* strains (1). All genes encoding SEs are harbored by various mobile elements. The complexity of enterotoxin gene regulation is not clear and requires further investigation, including full genomic sequences of enterotoxigenic *S. aureus* strains, which are the causative agents of SFP.

Here, we present the draft genome sequences of eight SE-producing *S. aureus* strains of clonal complexes 1 and 30 isolated during severe SFP outbreaks in the Russian Federation. *S. aureus* strains B-7738 and B-7739 were isolated from foodstuff samples and clinical specimens, respectively, during a widespread SFP outbreak among construction personnel in Saint Petersburg in 2013 (2). *S. aureus* strains B-7778 and B-7779 were isolated from clinic material and food handlers, respectively, during a large SFP outbreak at the International Youth Forum Seliger in 2014. *S. aureus* strains B-7904, B-7905, B-7906, and B-7907 were isolated from clinical specimens and foodstuff samples during an SFP outbreak among elementary school pupils and school personnel in Yakutsk in 2015. The production of enterotoxin A and/or enterotoxin B by isolated *S. aureus* strains was confirmed using the enzyme immunoassay Ridascreen set (R-Biopharm AG, Darmstadt, Germany).

*S. aureus* genomic DNA was extracted with cetyltrimethylammonium bromide (3). Whole-genome sequencing was done with the Ion Torrent Personal Genome Machine platform (Life Technologies, Inc.) using 250-bp read chemistry (strains B-7738 and B-7739) and 400-bp read chemistry (strains B-7778, B-7779, B-7904, B-7905, B-7906, and B-7907). Assembly of the sequence data was performed through Newbler 2.9 (strains B-7738, B-7739, B-7778, and B-7779) and SPAdes v. 3.8.1 (strains B-7904, B-7905, B-7906, and B-7907). Annotation was carried out using the NCBI Prokaryotic Genome Annotation Pipeline annotation pipeline (https://www.ncbi.nlm.nih.gov/genome/annotation_prok/). The MLST, VirulenceFinder, and PlasmidFinder (http://www.genomicepidemiology.org/) databases were used to find the sequence type, virulence genes, and the plasmid type, respectively, present in the sequenced genomes. Prophage regions were revealed using PHAge Search Tool (4). The results are summarized in Table 1.

**TABLE 1:** Genome characteristics of eight SE-producing *S. aureus* strains

Strain	GenBank accession no.	Draft genome size (bp)	No. of contigs	Coverage (×)	No. of CDS^a^	MGEs^b^	Genes associated with SFP^c^	ST/spa^d^
SCPM-O-B-7738	LWRA00000000	2,794,755	75	50	2,798	phiSa3-like, SaPI2, SaPI4, egc3	*sea*, *seg*, *sei*, *tst*	30/t2509
SCPM-O-B-7739	LWRB00000000	2,792,264	94	25	2,804	phiSa3-like, SaPI2, SaPI4, egc3	*sea*, *seg*, *sei*, *tst*	30/t2509
SCPM-O-B-7778	LWRC00000000	2,745,109	80	54	2,744	phiSa3-like, SaPI2, SaPI4, egc3	*sea*, *seg*, *sei*, *tst*	30/t122
SCPM-O-B-7779	LWRD00000000	2,750,534	72	112	2,744	phiSa3-like, SaPI2, SaPI4, egc3	*sea*, *seg*, *sei*, *tst*	30/t122
SCPM-O-B-7904	NIDA00000000	2,748,412	56	40	2,788	phiSa3-like, SaPI3, pMW2-like plasmid	*sea*, *seb*, *seh*	1/t948
SCPM-O-B-7905	NIDB00000000	2,704,426	40	72	2,720	SaPI3, pMW2-like plasmid	*seb*, *seh*	1/t948
SCPM-O-B-7906	NIDD00000000	2,700,794	48	28	2,715	SaPI3, pMW2-like plasmid	*seb*, *seh*	1/t948
SCPM-O-B-7907	NIDC00000000	2,752,673	93	23	2,804	phiSa3-like, SaPI3, pMW2-like plasmid	*sea*, *seb*, *seh*	1/t948

a CDS, coding sequences.

b MGE, mobile genetic element.

c *sea*, *seb*, *seg*, *sei*, and *seh*, genes of staphylococcal enterotoxin A, B, G, I, and H, respectively; *tst*, gene of toxic shock syndrome toxin.

d ST, sequence type; spa, *Staphylococcus aureus* protein A type.

The assembled genomes were approximately 2.7 Mb in length, with an average G+C content of ~32.7%. All strains except B-7905 and B-7906 harbored different complete phiSa3-like prophages encoding enterotoxin A. Strains B-7904, B-7905, B-7906, and B-7907 carried a pMW2-like penicillinase-encoding plasmid (about 20 kbp). BLAST comparative analysis highlighted various *S. aureus* pathogenicity islands (SaPI), such as SaPI2, SaPI3, SaPI4, and genomic island vSAβ with the enterotoxin gene cluster type egc3. All isolates were found to harbor various toxin genes associated with SFP (Table 1).

### Accession number(s).

The draft genome sequences have been deposited in DDBJ/ENA/GenBank under the accession numbers provided in Table 1.

## References

[1] Kadariya J, Smith TC, Thapaliya D 2014 *Staphylococcus aureus* and staphylococcal food-borne disease: an ongoing challenge in public health. Biomed Res Int 2014:827965. doi:10.1155/2014/827965. 24804250 PMC3988705

[2] Onishchenko GG, Abaev IV, Dyatlov IA, Skryabin YP, Korobova OV, Solovyov PV, Bogun AG 2014 Molecular genetic identification of *Staphylococcus aureus* strain, caused a foodborne illness outbreak in St. Petersburg in 2013. Vestn Ross Akad Med Nauk 33–38. doi:10.15690/vramn.v69i9-10.1129. 25816641

[3] Wilson K 2001 Preparation of genomic DNA from bacteria. Curr Protoc Mol Biol Chapter 2:Unit 2.4. doi:10.1002/0471142727.mb0204s56. 18265184

[4] Zhou Y, Liang Y, Lynch KH, Dennis JJ, Wishart DS 2011 PHAST: a fast phage search tool. Nucleic Acids Res 39:W347–W352. doi:10.1093/nar/gkr485. 21672955 PMC3125810

